# Effect of Cold Plasma and Plasma Activated Water on Polyphenols, Vitamins, and Volatile Compounds in Fresh‐Cut Carrots

**DOI:** 10.1002/fsn3.72526

**Published:** 2026-10-29

**Authors:** L. Acquaticci, E. Torregiani, J. B. Molina Hernandez, G. G. Gebremical, S. Tappi, G. Caprioli

**Affiliations:** ^1^ School of Pharmacy, Chemistry Interdisciplinary Project (ChIP) University of Camerino Camerino Italy; ^2^ Department of Agricultural and Food Sciences (DISTAL) University of Bologna Cesena Italy; ^3^ Interdepartmental Centre for Industrial Agri‐Food Research (CIRI) University of Bologna Cesena Italy

**Keywords:** antioxidant activity, bioactive compounds, plasma, plasma activated water, polyphenols, vitamins

## Abstract

This study aimed to evaluate content of bioactive compounds (polyphenols and vitamins B2 and B3 by HPLC‐MS/MS and spectrophotometric analysis, ascorbic acid by HPLC‐DAD and the volatile profile by HS‐SPME‐GC–MS) in fresh‐cut carrots treated with CP and PAW for 5‐10‐20‐30 min. The polyphenol content was affected mostly by PAW; the ascorbic acid content statistically increased by CP and PAW, especially after 10 min of treatment; nicotinamide decreased in both treatments, while nicotinic acid increased in CP‐treated samples. The volatile profile was also influenced by the application of CP and PAW: CP applied for 5 min slightly increased the abundance of volatile compounds whereas the PAW treatment completely changed the volatile profile. Finally, spectrophotometric analysis revealed the highest TPC value in CP‐treated carrots for 20 min and PAW‐treated for 5 min; the TFC value instead was higher in CP‐treated carrots for 5 min and with PAW for 20 min; antioxidant activity was higher in carrots treated with CP for 5 min and with PAW for 20 min. In conclusion, this study shows that both CP and PAW treatments contribute to altering the chemical composition of fresh‐cut carrots, with the PAW treatment having a greater impact on the content of some bioactive compounds, especially for polyphenolic compounds. In fact, a reduction of polyphenolic content of 29% and 26% was found in samples treated with CP for 10 and 30 min, respectively; while in PAW treated samples the reduction was around 70% regardless of the treatment time.

## Graphical abstract

Synthesis of the results of polyphenols, vitamins, and volatile compounds in fresh‐cut carrots treated with CP and PAW.

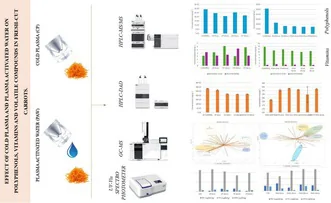

## 1 Introduction

Plasma is considered the fourth state of matter and it occurs in solid, liquid and gaseous form (Filipić and Dobnik 2020; Rathod and Annapure 2021). It is composed by a mixture of charged several particles, such as electrons or ions, and uncharged particles, such as atoms, molecules and radicals, as well as ultraviolet photons (Thirumdas and Valdramidis 2018).

Recently, the growing demand for healthy, fresh and safe food increased the interest in studying plasma as a potential alternative for the decontamination of food, as reported in several studies (Figure 1) (Hernández‐Hernández and Villanueva‐Rodríguez 2019; Deng and Kong 2005; Montenegro and Chen 2002; Niemira and Fridman 2005). One of the limits directly connected to the use of cold plasma for this purpose is the ineffective decontamination due to the irregular surface of food products, which can help microorganisms to hide in inaccessible areas (Thirumdas and Valdramidis 2018).

**FIGURE 1:**
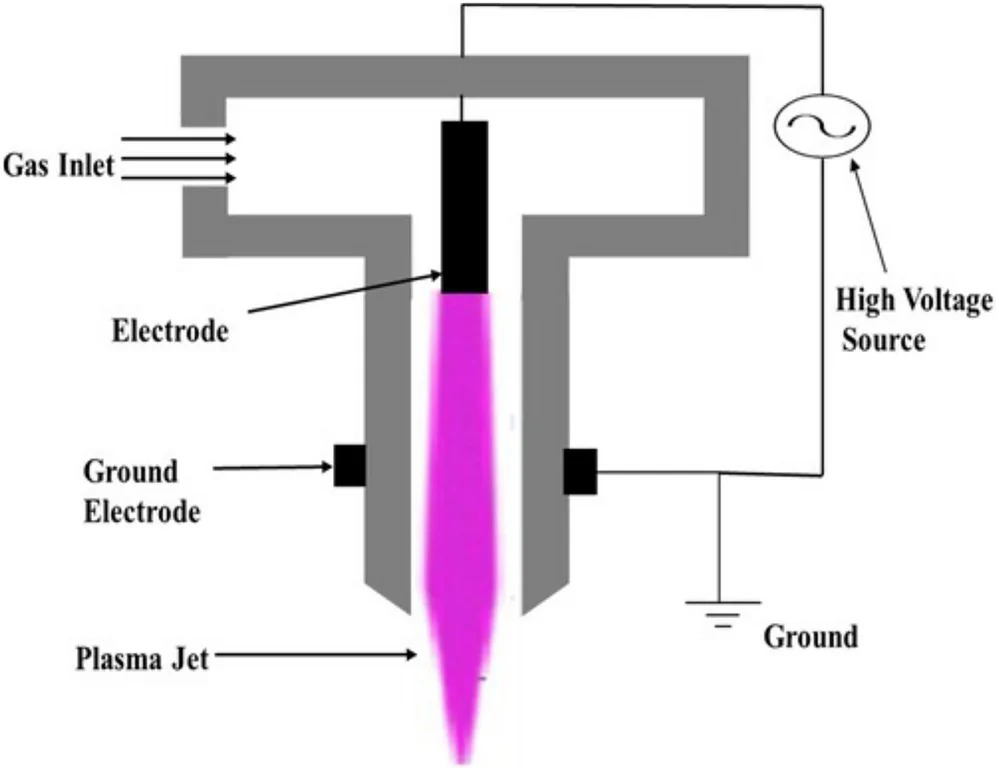
Cold plasma (CP) technology (Akhtar and Gebrekirstos 2022).

To overcome these problems, plasma activated water (PAW) has been developed and investigated as a promising alternative for food decontamination (Kaushik and Masur 2018; Thirumdas and Valdramidis 2018), as reported in several studied on fresh‐cut fruits (Chen and Hu 2019; Zhao and Song 2019; Liu and Hu 2020) and vegetables (Abouelenein and Vittori 2021). PAW can be generated by cold plasma in different ways (direct discharge within the liquids, discharges in the gas phase over the liquid surface, and multiphase discharges, such as discharges in bubbles inside liquids or in contact with liquid sprays or foams) (Figure 2) (Xiang and Bai 2022).

**FIGURE 2:**
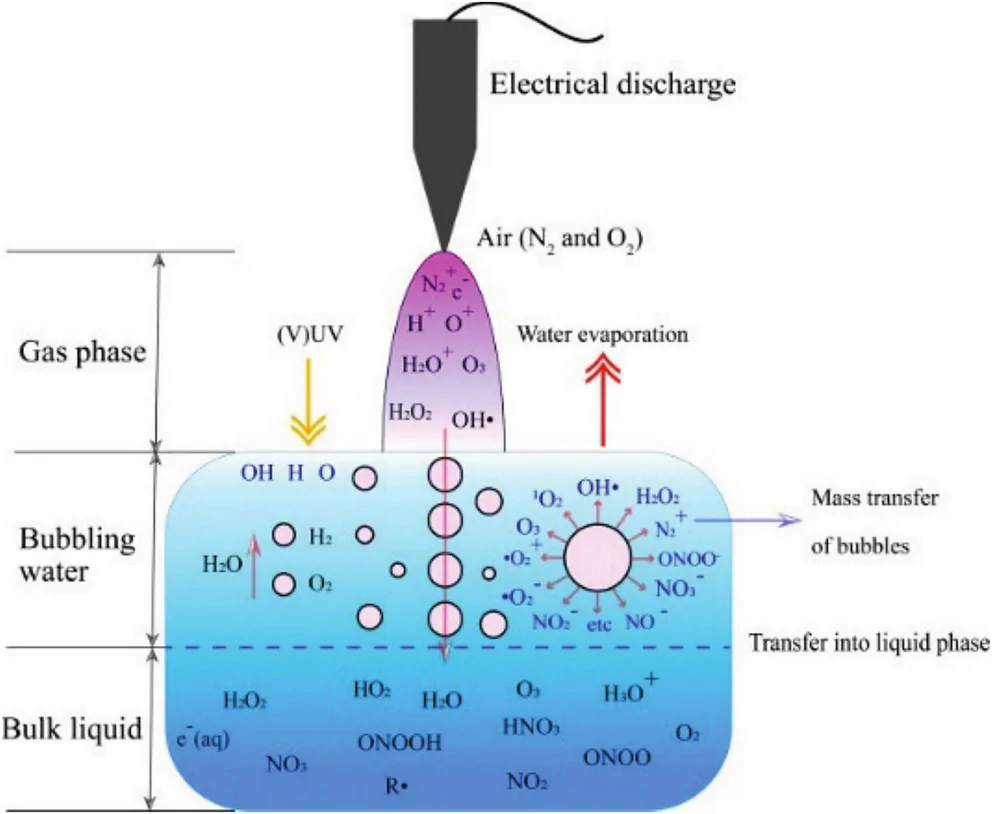
Plasma Activated Water (PAW) technology (Gao and Zhang 2022).

The demonstrated antimicrobial activity of plasma is mainly due to the presence of reactive oxygen/nitrogen species (ROS and RNS), which can cause oxidative damage to cellular lipids, proteins, and DNA of microorganisms but they can also react with macronutrients and bioactive molecules of food. These chemical reactions, such as nitration, nitrosation, hydroxylation, oxidation, sulfoxidation, deamidation, dehydrogenation or hydrogenation, and dimerization (Feizollahi and Roopesh 2020), can change the nutritional profile of the food product.

Moreover, studies on various foodstuff treated with CP showed opposite results regarding the content of different bioactive molecules, such as polyphenols and vitamins, suggesting a possible influence also dependent on the food product.

In this context, it is of great interest to analyze the effects of non‐thermal technologies (such as CP and PAW) on the nutritional profile of minimally processed foods, whose demand is constantly increasing.

The aim of this study was to determine the effect of CP and PAW on carrots by analyzing the content of polyphenols, ascorbic acid, vitamins B2 and B3 and volatiles by HPLC‐MS/MS, HPLC‐DAD, GC–MS and spectrophotometric analysis.

## 2 Materials and Methods

### 2.1 Chemicals and Reagents

Stock solutions (1000 mg·L^−1^) were prepared using analytical standard purchased form Sigma‐Aldrich (Milan, Italy). Each standard of polyphenolic compounds was dissolved in methanol (HPLC grade), ascorbic acid in 5% metaphosphoric acid (MPA), while nicotinic acid, nicotinamide and riboflavin were dissolved in water with acetic acid (2% *v*/*v*) and stored in the dark at 4°C until analysis.

Formic acid (99%) was purchased from Merck (Darmstadt, Germany), while deionized water (18 MΩ cm resistivity) was produced by a Milli‐Q SP Reagent Water System (Millipore, Bedford, MA, USA).

### 2.2 Samples

Whole fresh carrots ( *Daucus carota* ) were purchased from a local supermarket (Cesena, Italy), stored in a refrigerator at 5°C and used within 2 days. The average fruit mass was 478.55 ± 11.12 g, and the total soluble solids (TSS) content was around 13.00 °Brix.

Before processing, the whole carrots were washed with sodium hypochlorite (30 ppm for 5 min) and dried for 10 min. Then, each carrot was manually peeled and cut into “julienne” using a Vegetable Cutters (GAM International, TVEC UJMN230p‐ Igea Marina, ITA). All samples, approximately 80 g each, were freshly prepared the morning before treatment. For CP and PAW treatment, 2 separate batches of carrots were used.

### 2.3 PAW Treatment

PAW was prepared with 500 mL of sterile distilled water with a pulsed corona discharge for 1 min, driven by a high voltage generator (AlmaPulse, AlmaPlasma s.r.l., Bologna, Italy, maximum power: 600 W) with a maximum voltage of 15 kV and a pulse repetition frequency of 5 kHz (Gebremical and Romani 2023). The plasma was generated in ambient air for 60 s on the water surface in the air space (5 mm) between the tip of the stainless‐steel working electrode and the surface of the liquid. The pH value of PAW was 3.48 ± 0.08 and the measured concentrations of H_2_O_2_ and NO_2_ were 13.98 ± 3.47 mg/L, 1.18 ± 0.06 mg/L, respectively.

Different washing times for freshly prepared PAW were tested (5, 10, 20, and 30 min). The ratio of carrots to liquid was kept constant (1:3 w/v). The treatments (two replicates each) were performed on an oscillating shaker; no acidified‐water control was included, so the individual contribution of PAW's acidic pH versus its reactive plasma species to the observed changes cannot be isolated.

### 2.4 Cold Plasma Treatment

For the gaseous CP treatment, the system used was previously described (Molina‐Hernandez and Chaves‐López 2022, 2023), but for the sake of completeness, the most important details are reported. A Surface Dielectric Barrier Discharge (SDBD) plasma source connected to a high voltage generator (Alma Pulse, Alma Plasma s.r.l., Bologna, Italy, maximum power: 600 W) and placed on top of a treatment chamber (internal volume: 18.5 L, height: 20 cm) was used. The SDBD consists of 4 rectangular high‐voltage electrodes (115 cm^2^ each), in which the dielectric material is represented by a 2 mm thick mica layer. A sinusoidal waveform with a peak voltage of 6 kV and a repetition frequency of 23 kHz was obtained by the generator; while the plasma source absorbed 425.35 ± 25.79 W of power density, resulting in a surface power density of 2.6 W/cm^2^ and a transitional NOx regime. Air was used for plasma generation. The fresh‐cut carrots were placed on a stainless‐steel tray at a distance of 4 cm from the surface of the SDBD and subjected to CP treatment for 5, 10, 20, and 30 min. The treatments were carried out at room temperature (26°C ± 1°C). After 30 min, the maximum concentration of NOx reached was 1690 ± 248 ppm. Each treatment was repeated twice in two separate experiments. The untreated sample was considered as control.

### 2.5 Analysis of Polyphenols by HLPC‐MS/MS

#### 2.5.1 Extraction of Polyphenols

100 mg of freeze‐dried carrots were extracted with 10 mL of 70% ethanolic solution (acidified with 1.5% of formic acid) in an ultrasound bath for 60 min at 25°C. After centrifugation, the supernatant was recovered, while the residue was washed with other 5 mL of 70% ethanolic solution (acidified with 1.5% of formic acid). The solution was centrifuged; the supernatant was recovered and evaporated with rotavapor. Then, the residue was lyophilized to remove water and finally dissolved in 5 mL of methanol. The sample was filtered before the injection using a Phenex RC, 4‐mm, 0.2‐μm syringeless filter from Phenomenex (Castel Maggiore, BO, Italy). The extraction was repeated in duplicates.

#### 2.5.2 HPLC‐MS/MS Analysis

An Agilent 1290 Infinity series coupled with Triple Quadropole 6420 from Agilent Technology (Santa Clara, CA, USA) was used to quantify 36 polyphenolic compounds with a method previously developed by Mustafa and Caprioli (2022). The separation was performed by a Synergi Polar–RP C18 analytical column (250 mm × 4.6 mm, 4 μm) from Phenomenex (Chesire, UK) and a gradient elution with water and methanol both acidified with 0.1% of formic acid at a flow rate of 0.8 mL·min^−1^.

The electrospray ionization (ESI) source operated in both positive and negative modes and the transitions for each compound, used for qualification and quantification, were monitored in dynamic‐multiple reaction monitoring (dynamic‐MRM) mode, as reported in Table S1.

### 2.6 Analysis of Ascorbic Acid

#### 2.6.1 Extraction of Ascorbic Acid

100 mg of freeze‐dried sample was extracted with 5 mL of an aqueous solution of 5% meta‐phosphoric acid (MPA) in the darkness under stirring. After centrifugation, the supernatant was collected and the precipitate was washed with other 5 mL of 5% MPA and centrifuged for 10 min at 5000 rpm. The supernatant was filtered using a 0.45 μm membrane filter before the injection in HPLC. The extraction was repeated in duplicates.

#### 2.6.2 Analysis of Ascorbic Acid

Ascorbic acid was analyzed by a Hewlett Packard (Palo Alto, CA, USA) HP‐1090 Series II coupled with a diode‐array detector (DAD) and a Synergi Polar‐RP C18 (4.6 mm × 150 mm, 4 μm) analytical column from Phenomenex (Chesire, UK) with an isocratic elution (Caprioli and Sagratini 2014). The mobile phase was composed by 90% of water and 10% of methanol both acidified with 0.1% formic acid and a flow rate of 0.5 mL·min^−1^. UV spectra were recorded from 210 to 400 nm wavelengths and 245 nm was used for quantification.

### 2.7 Analysis of Riboflavin, Nicotinamide and Nicotinic Acid

#### 2.7.1 Extraction of Riboflavin, Nicotinamide and Nicotinic Acid

The extraction of vitamins was conducted by stirring 200 mg of lyophilized sample with 10 mL of 0.1 N hydrochloric acid (HCl) at 100°C for 30 min. Then, the pH was adjusted to 4–4.5 by adding 2 M sodium acetate solution and after centrifugation and filtration the extract was analyzed in HPLC‐MS/MS. The extraction was repeated in duplicates.

#### 2.7.2 Analysis of Riboflavin, Nicotinamide and Nicotinic Acid by HPLC/MS–MS

An Agilent 1290 Infinity series coupled with a Triple Quadrupole 6420 from Agilent Technology (Santa Clara, CA, USA) with an electrospray (ESI) source working in positive mode was used to analyze vitamins. A Kinetex Hilic analytical column (100 mm 4.6 mm i.d., particle size 2.6 μm) from Phenomenex with a mobile phase of 95% water and 5% acetonitrile both acidified with 0.1% formic acid in isocratic conditions at a flow rate of 0.8 mL·min^−1^ were used to perform the separation (Caprioli and Torregiani 2018). Transitions were monitored in dynamic‐multiple reaction monitoring (dynamic‐MRM) mode and used for the qualification and the quantification, as reported in Table S2.

### 2.8 Analysis of Volatile Compounds by GC–MS

#### 2.8.1 Headspace‐Solid Phase‐Microextraction (HS‐SPME)

HS‐SPME was used to extract volatiles from carrots. Briefly, 200 mg of sample were placed in a 20 mL vial, tightly clapped with a PTFE/silicon septum, incubated for 15 min at 60°C under stirring and then extracted for 30 min with a divinylbenzene/carbon‐wide range/polydimethylsiloxane (DVB/C‐WR/PDMS) 80 μm fiber. After the extraction, the fiber was inserted into the injector port at a speed of 100 mm·sec^−1^ and a penetration depth of 40 mm at 250°C for 3 min for the desorption. The extraction was repeated in duplicates.

#### 2.8.2 Gas Chromatography–Mass Spectrometry (GC–MS)

Volatile compounds were analyzed through a GC–MS, composed by an Agilent 8890 GC equipped with a PAL RTC 120 autosampler and coupled to an Agilent 5977B MSD quadrupole detector with an electron ionization (EI) source (Santa Clara, California, USA).

The injector temperature was set at 250°C, and the liner used was recommended for SPME injection, namely, Inlet liner, Ultra Inert, splitless, straight, 0.75 mm id, from Agilent. The gas carrier was helium at flow rate of 1 mL min^−1^.

The separation of volatiles was performed with a DB‐WAX capillary column (60 m, 250 μm i.d., 0.25 μm film thickness) with a gradient temperature program (initial temperature at 35°C, then increased to 60°C at a rate of 15°C/min, then increased to 115°C at 5°C/min, then to 200°C at 2°C/min and finally 250°C at 5°C/min and held for 5 min). The acquisition was performed in SCAN mode (35–450 m/z) and peak identification was performed by comparing the mass spectrum and experimental linear retention index (RI) with data from the NIST library (US National Institute of Standards and Technology) and those reported in literature. The data results were managed by the MSD ChemStation software (Agilent, version G1701DA D.01.00).

### 2.9 Spectrophotometric Analysis

#### 2.9.1 Antioxidant Activity (DPPH)

DPPH method was used to determine the antioxidant activity. Briefly, 0.5 mL of ethanolic extract was mixed with 4.5 mL of 0.1 mM ethanolic solution of DPPH and left in the dark for 30 min (Mustafa and Caprioli 2022). After that, the oxidation of 2,2‐diphenyl‐1‐picrylhydrazyl (DPPH) was measured spectrophotometrically at 517 nm and results were reported as mg trolox equivalent (TE) per kg sample (mg TE/kg). The analysis was repeated in triplicates.

#### 2.9.2 Total Phenolic Content (TPC)

TPC was determined spectrophotometrically at 700 nm in a solution obtained by the reaction of 0.5 mL of ethanolic extract with 2.5 mL of Folin–Ciocalteu solution and 7 mL of Na_2_CO_3_ (7.5% aqueous solution), left for 2 h in dark at room temperature. Results were expressed as mg of gallic acid equivalents (GAE) per kg sample (mg GAE/kg). The analysis of samples was repeated in triplicates.

#### 2.9.3 Total Flavonoid Content (TFC)

TFC was determined by mixing 0.5 mL of ethanolic extract with 0.15 mL of 0.5 M NaNO_2_, 3.2 mL of 30% methanol, 0.15 mL of 0.3 M AlCl_3_ and 1 mL of 1 M NaOH. After 30 min of incubation in the dark at room temperature the absorbance was measures spectrophotometrically at 506 nm and results were expressed as mg of rutin equivalents (RE) per kg sample (mg RE/kg). The analysis of samples was repeated in triplicates.

### 2.10 Statistical Analysis

All the obtained results were expressed as mean value ± standard deviation. Significant differences between data were calculated by unidirectional analysis of variance (ANOVA) followed by Tukey's post hoc comparison test, with a significance level of *p* < 0.05. The statistical package STATISTICA 8.0 software (StatSoft, Tulsa, UK) was used.

Data on volatile compounds were examined by principal component analysis (PCA) using Statistica v.7.1 (Stat Soft Italia, Vigonza, Italy). One covariance data matrix was created: it included 59 variables and 10 samples (volatiles and different carrot samples). The data were analyzed using principal component analysis (PCA) with STATISTICA 7.1 (Stat Soft Italia srl, 2005, www.statsoft.it).

## 3 Results

### 3.1 Analysis of Polyphenols Through HPLC‐MS/MS

Polyphenols are known for their strong antioxidant and anti‐inflammatory activity; for this reason, it's important to monitor their concentration in food to keep a state of good‐health. The results of the polyphenol content (mg·kg^−1^) are reported in Table 1 (for CP‐treated carrots) and Table 2 (for PAW‐treated carrots). Gallic, neochlorogenic, chlorogenic, 4‐hydroxybenzoic, caffeic, *p*‐coumaric, ferulic, ellagic acids, delphinidin‐3,5‐diglucoside, phloridzin, quercetin and kaempferol were the polyphenols found in the carrots. Generally, the concentration of polyphenols decreased with increasing treatment duration, with statistically significant differences in both CP and PAW treatments.

**TABLE 1:** Polyphenolic content (mg·kg^−1^) in carrots treated with cold plasma (CP).

CP	Control	5 min	10 min	20 min	30 min
Gallic acid	n.d.	n.d.	n.d.	n.d.	n.d.
Neochlorogenic acid	0.7 ± 0.00^a^	n.d.	0.7 ± 0.00^a^	0.7 ± 0.02^a^	n.d.
Delphinidin‐3‐galactoside	n.d.	n.d.	n.d.	n.d.	n.d.
Catechin	n.d.	n.d.	n.d.	n.d.	n.d.
Procyanidin B2	n.d.	n.d.	n.d.	n.d.	n.d.
Chlorogenic acid	23.1 ± 2.98^a^	19.2 ± 0.24^ab^	15.3 ± 1.39^b^	19.1 ± 1.42^ab^	16.1 ± 0.38^b^
4‐Hydroxy benzoic acid	4.9 ± 0.00^a^	4.9 ± 0.01^a^	5.1 ± 0.02^b^	5.1 ± 0.00^b^	5.1 ± 0.04^b^
Epicatechin	n.d.	n.d.	n.d.	n.d.	n.d.
Cyanidin‐3‐glucoside	n.d.	n.d.	n.d.	n.d.	n.d.
Petunidin‐3‐glucoside	n.d.	n.d.	n.d.	n.d.	n.d.
3‐Hydroxy benzoic acid	n.d.	n.d.	n.d.	n.d.	n.d.
Caffeic acid	n.d.	n.d.	n.d.	n.d.	n.d.
Vanillic acid	n.d.	n.d.	n.d.	n.d.	n.d.
Pelargonidin‐3‐glucoside	n.d.	n.d.	n.d.	n.d.	n.d.
Pelargonidin‐3‐rutinoside	n.d.	n.d.	n.d.	n.d.	n.d.
Malvidin‐3‐galactoside	n.d.	n.d.	n.d.	n.d.	n.d.
Syringic acid	n.d.	n.d.	n.d.	n.d.	n.d.
Procyanidin A2	n.d.	n.d.	n.d.	n.d.	n.d.
*p*‐Coumaric acid	0.49 ± 0.00^a^	0.5 ± 0.01^a^	n.d.	0.5 ± 0.00^a^	0.5 ± 0.01^a^
Ferulic acid	n.d.	n.d.	n.d.	n.d.	n.d.
Rutin	n.d.	n.d.	n.d.	n.d.	n.d.
Hyperoside	n.d.	n.d.	n.d.	n.d.	n.d.
Isoquercitrin	n.d.	n.d.	n.d.	n.d.	n.d.
Delphinidin‐3,5‐diglucoside	n.d.	n.d.	n.d.	n.d.	n.d.
Phloridzin	n.d.	n.d.	n.d.	0.1 ± 0.01^a^	0.1 ± 0.01^a^
Quercitrin	n.d.	n.d.	n.d.	n.d.	n.d.
Myricetin	n.d.	n.d.	n.d.	n.d.	n.d.
Naringin	n.d.	n.d.	n.d.	n.d.	n.d.
Kaempferol‐3‐glucoside	n.d.	n.d.	n.d.	n.d.	n.d.
Hesperidin	n.d.	n.d.	n.d.	n.d.	n.d.
Ellagic acid	n.d.	n.d.	n.d.	n.d.	n.d.
Quercetin	n.d.	n.d.	n.d.	n.d.	n.d.
Phloretin	n.d.	n.d.	n.d.	n.d.	n.d.
Kaempferol	n.d.	n.d.	n.d.	n.d.	n.d.
Isorhamnetin	n.d.	n.d.	n.d.	n.d.	n.d.
Total	29.28^a^	24.68^ab^	21.07^b^	25.53^ab^	21.83^b^

*Note:* Different letters indicate statistically significant differences (*p* < 0.05) among samples with the same treatment (*n* = 2).

Abbreviation: n.d., Not detected.

**TABLE 2:** Polyphenolic content (mg·kg^−1^) in carrots treated with plasma activated water (PAW).

PAW	Control	5 min	10 min	20 min	30 min
Gallic acid	0.9 ± 0.04^a^	0.5 ± 0.00^c^	0.3 ± 0.02^d^	0.4 ± 0.01^cd^	0.3 ± 0.01^d^
Neochlorogenic acid	n.d.	0.8 ± 0.01^a^	0.7 ± 0.01^b^	0.7 ± 0.75^b^	n.d.
Delphinidin‐3‐galactoside	n.d.	n.d.	n.d.	n.d.	n.d.
Catechin	n.d.	n.d.	n.d.	n.d.	n.d.
Procyanidin B2	n.d.	n.d.	n.d.	n.d.	n.d.
Chlorogenic acid	2.2 ± 0.30^a^	1.8 ± 0.05^ac^	1.3 ± 0.04^c^	0.2 ± 0.04^d^	1.1 ± 0.04^c^
4‐Hydroxy benzoic acid	5.2 ± 0.08^a^	5.2 ± 0.03^a^	5.1 ± 0.06^a^	5.1 ± 0.03^a^	5.2 ± 0.01^a^
Epicatechin	n.d.	n.d.	n.d.	n.d.	n.d.
Cyanidin‐3‐glucoside	n.d.	n.d.	n.d.	n.d.	n.d.
Petunidin‐3‐glucoside	n.d.	n.d.	n.d.	n.d.	n.d.
3‐Hydroxy benzoic acid	n.d.	n.d.	n.d.	n.d.	n.d.
Caffeic acid	2.8 ± 0.02^ab^	2.7 ± 0.00^ab^	2.8 ± 0.13^ab^	2.58 ± 0.01^b^	2.7 ± 0.08^ab^
Vanillic acid	n.d.	n.d.	n.d.	n.d.	n.d.
Pelargonidin‐3‐glucoside	n.d.	n.d.	n.d.	n.d.	n.d.
Pelargonidin‐3‐rutinoside	n.d.	n.d.	n.d.	n.d.	n.d.
Malvidin‐3‐galactoside	n.d.	n.d.	n.d.	n.d.	n.d.
Syringic acid	n.d.	n.d.	n.d.	n.d.	n.d.
Procyanidin A2	n.d.	n.d.	n.d.	n.d.	n.d.
*p*‐Coumaric acid	1.4 ± 0.12^a^	1.3 ± 0.05^a^	1.2 ± 0.01^a^	1.2 ± 0.03^a^	0.8 ± 0.03^b^
Ferulic acid	4.0 ± 0.35^a^	3.8 ± 0.01^a^	3.7 ± 0.01^a^	3.7 ± 0.03^a^	3.7 ± 0.02^a^
Rutin	n.d.	n.d.	n.d.	n.d.	n.d.
Hyperoside	n.d.	n.d.	n.d.	n.d.	n.d.
Isoquercitrin	0.5 ± 0.00	n.d.	n.d.	n.d.	n.d.
Delphinidin‐3,5‐diglucoside	1.00 ± 0.00	n.d.	n.d.	n.d.	n.d.
Phloridzin	n.d.	n.d.	n.d.	n.d.	n.d.
Quercitrin	n.d.	n.d.	n.d.	n.d.	n.d.
Myricetin	n.d.	n.d.	n.d.	n.d.	n.d.
Naringin	n.d.	n.d.	n.d.	n.d.	n.d.
Kaempferol‐3‐glucoside	n.d.	n.d.	n.d.	n.d.	n.d.
Hesperidin	n.d.	n.d.	n.d.	n.d.	n.d.
Ellagic acid	33.0 ± 0.01^a^	n.d.	n.d.	n.d.	n.d.
Quercetin	0.4 ± 0.03^a^	n.d.	n.d.	n.d.	n.d.
Phloretin	n.d.	n.d.	n.d.	n.d.	n.d.
Kaempferol	n.d.	n.d.	n.d.	n.d.	n.d.
Isorhamnetin	n.d.	n.d.	n.d.	n.d.	n.d.
TOTAL	51.64^a^	16.14^b^	15.65^b^	14.37^b^	14.45^b^

*Note:* Different letters indicate statistically significant differences (*p* < 0.05) among samples with the same treatment (*n* = 2).

Abbreviation: n.d., Not detected.

Since the CP and PAW treatments were not applied at the same batch of carrots, concentrations could not be compared directly but the percentage of variation of chemical composition with respect to each control could have been calculate to compare results.

In the CP treated carrots, the total content of polyphenols decreased as the time of treatment increased, with statistically significant differences in the carrots treated for 10 and 30 min with respect to the control. In fact, after 10 and 30 min of treatment, there was a reduction in the polyphenolic content of 29% and 26%, respectively.

The decrease in total polyphenol content was more effective in the PAW treated carrots; in fact, the polyphenol content was statistically lower in the PAW treated carrots (14.37–16.14 mg·kg^−1^) with respect to the control (51.64 mg·kg^−1^), while the differences were less remarkable among different times of treatment (not statistically different). In fact, there was a decrease in polyphenols by about 70% regardless of the treatment time. Gallic acid, in particular, drastically decreased in carrots treated with PAW with respect to the control. This result is in contrast to data from the literature reporting an increased concentration of this compound in strawberry juice, fresh‐cut pitaya fruit and green tea leaves treated with cold plasma (Rana and Yadav 2020; Li and Li 2019; Keshavarzi and Ghomi 2020). Neochlorogenic acid was mainly influenced by the application of PAW; in fact, its concentration increased after 5 min of treatment, while it remained unchanged in the samples treated for 10 and 20 min.

Chlorogenic acid was found to be the most abundant in the carrots treated with CP. One study reported that the concentration of chlorogenic acid in the samples generally increased by 39.85% and 46.5% after 10–15 min of treatment, respectively. However, when the treatment duration was increased to 30 min, the concentration remained at 35% (Rana and Yadav 2020). A similar increase in the concentration of chlorogenic acid was reported by Herceg and Dragović‐Uzelac (2016) in pomegranate juice. Chlorogenic acid in plasma‐treated pomegranate juice increased from 2.1 to 4.95 mg·mL^−1^ after plasma treatment. The results about chlorogenic acid concentration were opposite in this study: in both CP and PAW‐treated carrots, its concentration decreased with the application of the treatment and with the extension of the treatment duration. Specifically, for the CP treated carrots, the decrease was statistically significant in the samples treated for 10–30 min, while in the PAW treated carrots the decrease was statistically significant after 10, 20, and 30 min.

4‐Hydroxybenzoic acid did not undergo any transformation after both treatments, as well as caffeic acid, which was only present in the PAW treated carrots. Caffeic acid was found to increase in PAW‐treated arugula samples for 20 min, while its concentration decreased in plasma‐treated pitaya fruits, lettuce leaves and pomegranate juice (Grzegorzewski and Rohn 2011; Herceg and Dragović‐Uzelac 2016). The concentration of *p*‐coumaric acid remained almost constant in all the samples treated with CP, except for the samples treated for 10 min. In the PAW‐treated sample, the concentration of *p*‐coumaric acid decreased with increasing treatment time, without statistically significant differences, except for the samples treated for 30 min.

Ferulic acid decreased slightly with the increase of PAW time treatment in carrots, although there were no statistically significant differences. A similar trend was also reported in arugula samples treated with PAW for different durations (Abouelenein and Vittori 2022): ferulic acid content decreased with increasing treatment time, with statistically significant differences only after 30 min. Delphinidin‐3‐glucoside, ellagic acid and quercetin completely disappeared after the treatment with PAW, regardless of the duration of treatment. In this case, the degradation of anthocyanins after plasma treatments was reported in several studies in the literature. For example, plasma treatment reduced the anthocyanin content in chokeberry and blueberry juice with increasing tension and treatment duration (Lacombe and Chen 2015; Sarangapani and Bourke 2017). The reduction in anthocyanin content was attributed to the oxidative cleavage of chromophores by hydroxyl radicals and ozone (Lacombe and Chen 2015).

Ellagic acid and quercetin decreased with increasing treatment time in PAW‐treated arugula, but without statistically significant differences; the only exception was the PAW‐treated arugula for 30 min, in which the content quercetin increased (Abouelenein and Vittori 2022). Regarding CP treatment, quercetin was reported not to undergo significant variations in onion samples (Kim and Min 2017).

### 3.2 Analysis of Ascorbic Acid

Ascorbic acid, or vitamin C, was quantified in samples of carrots treated with CP and PAW using a quantitative method by HPLC‐DAD.

Vitamin C is known for its antioxidant activity, which protects proteins, lipids and DNA from oxidative damage caused by free radicals or reactive species (de Castro and Campelo 2020). It is often used by researchers as food quality indicator, due to its high sensitivity to environmental changes (Barba and Frígola 2012). The results showed that the CP treatment affected the ascorbic acid content (Table 3). In particular, the concentration of ascorbic acid decreased with increasing treatment duration with statistically significant differences up to 10 min. Thereafter, the concentration decreased, even if the treatment duration increased, without statistically significant differences. This result was in contrast to the results found in the literature: in fact, plasma treatment was usually reported to increase the concentration of several bioactive compounds, including ascorbic acid (Castro and Campelo 2020; Kim and Min 2017; Paixão and Rodrigues 2019; Song and Fan 2020). The influence of these treatments on the content of bioactive compounds depends on the type of food treated, as reported in the literature. In the case of PAW treated carrots, on the other hand, the treatment did not seem to have a significant impact on ascorbic acid content (Table 3). In fact, the concentration of ascorbic acid increased slightly after 5–10 min of treatment, then decreased slightly after 20 min and it rose again after 30 min. In any case, the differences in concentration over the various treatment times were not statistically significant. A decrease in the content of ascorbic acid in the roots of tomato seedlings was observed in samples treated with PAW for 15 and 30 min (Adhikari and Choi 2019) and in orange juice treated with PAW (Xu and Keener 2017).

**TABLE 3:** Concentration (mg·kg^−1^) of ascorbic acid in carrots treated with cold plasma (CP) and plasma activated water (PAW).

	Control	5 min	10 min	20 min	30 min
CP	282.65 ± 3.11^a^	263.20 ± 8.14^b^	219.56 ± 0.75^c^	215.35 ± 0.84^c^	216.86 ± 1.72^c^
PAW	182.52 ± 0.25^a^	198.02 ± 0.40^a^	196.24 ± 0.38^a^	189.61 ± 10.70^a^	197.25 ± 0.97^a^

*Note:* Different letters indicate statistically significant differences (*p* < 0.05) among samples with the same treatment (*n* = 2).

### 3.3 Analysis of Riboflavin, Nicotinamide and Nicotinic Acid

Riboflavin (Vitamin B2), nicotinamide and nicotinic acid (or niacin) were analyzed using a method previously published (Caprioli and Torregiani 2018). Nicotinamide and nicotinic acid are the two main forms of vitamin B3. The vitamin content in different samples of carrots treated with CP and PAW is shown in Tables 4 and 5, respectively. While riboflavin was completely absent in all samples, the content of vitamin B3 was influenced by the CP and PAW treatments. Vitamin B3 is composed of two main forms, nicotinic acid (or niacin) and nicotinamide, which have the same vitaminic activity. In fact, the concentration of vitamin B3, which corresponded to the sum of the concentrations of nicotinamide and nicotinic acid, generally increased with CP treatment, while it generally decreased with PAW treatment. Specifically, with regard to the CP treatment on nicotinamide content, the initial concentration of 4.28 mg·kg^−1^ decreased up to 10 min of treatment, even if with no statistically significant differences. The highest concentration was reached after 20 min of treatment, while the lowest one after 30 min. Regarding nicotinic acid, the highest value was found in the samples treated with CP for 10 min, with statistically significant differences from the other samples. On the other hand, the lowest value was found in carrots treated for 20 min. This result was also reported in the literature: in fact, the concentration of nicotinic acid resulted to be enhanced by 20% by 10 min of CP treatment in Siriguela juice (Paixão and Rodrigues 2019). The content of nicotinamide increased until 10 min of PAW treatment, even if with no statistically significant differences. In contrast, its content in carrots treated for 20 and 30 min decreased with no statistically significant differences. On the other hand, the concentration of nicotinic acid generally decreased when the treatment time increased in the PAW treated carrots with no statistically significant differences. These results are consistent with those from the literature, which found no statistically significant differences in the nicotinamide content in rocket‐salad samples treated with PAW (Abouelenein and Vittori 2022). On the contrary, the results of nicotinic acid content were not consistent with those in the literature, which found that nicotinic acid increased in rocket‐leaves samples treated with PAW for 20 min (Abouelenein and Vittori 2022).

**TABLE 4:** Concentration (mg·kg^−1^) of Riboflavin, Nicotinamide, and Nicotinic acid in carrots treated with cold plasma (CP).

CP	Control	5 min	10 min	20 min	30 min
Riboflavin	n.d.	n.d.	n. d.	n.d.	n.d.
Nicotinamide	4.28 ± 0.83^a^	3.22 ± 0.56^a^	3.50 ± 0.95^a^	5.43 ± 0.40^a^	3.19 ± 0.62^a^
Nicotinic ACID	3.16 ± 0.62^a^	4.24 ± 0.99^b^	6.21 ± 0.78^c^	3.03 ± 0.21^a^	5.85 ± 0.00^d^
SUM (Nicotinic acid and Nicotinamide)	7.44	7.46	9.71	8.46	9.04

*Note:* Different letters indicate statistically significant differences (*p* < 0.05) among samples with the same treatment (*n* = 2).

**TABLE 5:** Concentration (mg·kg^−1^) of Riboflavin, Nicotinamide, and Nicotinic acid in carrots treated with plasma activated water (PAW).

PAW	Control	5 min	10 min	20 min	30 min
Riboflavin	n.d.	n.d.	n.d.	n.d.	n.d.
Nicotinamide	0.87 ± 0.02^a^	1.00 ± 0.01^a^	1.03 ± 0.13^a^	0.73 ± 0.12^a^	0.83 ± 0.19^a^
Nicotinic acid	13.68 ± 0.37^a^	11.93 ± 0.36^a^	10.86 ± 1.74^a^	10.06 ± 0.55^a^	10.70 ± 1.19^a^
SUM (Nicotinic acid and Nicotinamide)	14.55	12.93	11.89	10.79	11.53

*Note:* Different letters indicate statistically significant differences (*p* < 0.05) among samples with the same treatment (*n* = 2).

### 3.4 Analysis of Volatile Compounds by HS‐SPME‐GC‐MS

The volatile profile can be influenced by different plasma treatment parameters: type of plasma source, equipment and process parameters, reactive species and matrix (Abouelenein and Mustafa 2023). Generally, plasma treatment is associated with an increase in fat oxidation in the food product, which lead to the production of secondary metabolites such as aldehydes, alcohols, furans and hydrocarbons (Wojtasik‐Kalinowska and Poltorak 2023).

Different chemical classes were identified in carrots treated with CP and PAW: aldehydes, alcohols, terpenes, esters, furans, ketones, hydrocarbons, organic acids, nitriles and carotenoids (Tables 6 and 7).

**TABLE 6:** Volatile composition (% area) of carrots treated with cold plasma (CP).

	R.I.	Control	5 min	10 min	20 min	30 min
*Aldehydes*						
Heptanal	1194	2.14 ± 0.01	1.87 ± 0.27	1.12 ± 0.21	1.30 ± 0.02	1.22 ± 0.05
2‐Hexenal	1228	0.64 ± 0.75	0.69 ± 0.08	n.d.	0.34 ± 0.01	0.32 ± 0.02
Octanal	1291	4.94 ± 0.51	4.60 ± 0.06	4.11 ± 0.03	3.65 ± 0.05	3.58 ± 0.22
2‐Heptenal	1332	3.10 ± 0.32	2.86 ± 0.21	2.18 ± 0.09	2.62 ± 0.06	2.51 ± 0.32
Nonanal	1396	0.95 ± 0.51	1.11 ± 0.03	2.20 ± 0.40	1.09 ± 0.08	0.96 ± 0.06
2‐Octenal	1434	6.25 ± 0.14	6.25 ± 0.09	3.82 ± 0.30	5.42 ± 0.16	5.28 ± 0.31
2,4‐Heptadienal	1497	n.d.	0.27 ± 0.02	0.17 ± 0.00	0.52 ± 0.05	0.48 ± 0.01
Decanal	1502	0.26 ± 0.11	0.57 ± 0.06	n.d.	n.d.	n.d.
Benzaldehyde	1528	0.70 ± 0.31	0.71 ± 0.06	1.52 ± 0.28	1.71 ± 0.01	1.57 ± 0.11
Benzeneacetaldehyde	1638	n.d.	0.66 ± 0.06	1.67 ± 0.33	0.60 ± 0.10	0.51 ± 0.10
2‐Decenal	1645	n.d.	0.47 ± 0.00	0.24 ± 0.00	n.d.	0.21 ± 0.00
2‐Butyl‐2‐octenal	1653	n.d.	0.25 ± 0.03	0.31 ± 0.05	0.40 ± 0.03	0.27 ± 0.01
2,4‐Nonadienal	1705	1.78 ± 0.33	1.79 ± 0.13	1.34 ± 0.25	1.92 ± 0.04	1.93 ± 0.07
2,4‐Dimethyl‐benzaldehyde	1710	n.d.	n.d.	0.15 ± 0.00	0.25 ± 0.03	n.d.
2,4‐Decadienal	1808	n.d.	0.13	0.10 ± 0.00	0.27 ± 0.01	0.26 ± 0.00
Tot %		20.76	22.23	18.93	20.09	19.10
*Alcohols*						
1‐Pentanol	1250	3.62 ± 0.06	3.59 ± 0.41	1.45 ± 0.00	2.89 ± 0.25	2.64 ± 0.23
1‐Hexanol	1352	1.67 ± 0.07	2.41 ± 0.00	1.43 ± 0.20	1.33 ± 0.00	1.26 ± 0.01
1‐Octen‐3‐ol	1445	3.84 ± 0.06	3.45 ± 0.04	3.20 ± 0.62	4.18 ± 0.07	3.67 ± 0.06
1‐Heptanol	1455	0.67 ± 0.02	0.63 ± 0.03	n.d.	n.d.	n.d.
Linalool oxide	1480	n.d.	0.33 ± 0.024	n.d.	n.d.	n.d.
2,3‐Butanediol	1576	n.d.	0.61 ± 0.00	0.20 ± 0.29	n.d.	0.33 ± 0.06
*p*‐Cymen‐8‐ol	1846	0.50 ± 0.00	0.41 ± 0.00	0.79 ± 0.10	0.60 ± 0.00	0.58 ± 0.01
Benzyl alcohol	1865	n.d.	n.d.	0.40 ± 0.05	n.d.	n.d.
Phenylethyl alcohol	1946	n.d.	n.d.	n.d.	0.58 ± 0.01	0.48 ± 0.03
Tot %		10.30	11.43	7.47	9.58	8.96
*Terpenes*						
Sabinene	1133	n.d.	0.26 ± 0.00	n.d.	n.d.	n.d.
Limonene	1205	1.63 ± 0.25	n.d.	0.36 ± 0.07	n.d.	n.d.
*p*‐Cymene	1250	1.17 ± 0.10	0.98 ± 0.05	0.38 ± 0.00	n.d.	n.d.
*m*‐Cymene	1269	n.d.	n.d.	0.57 ± 0.00	0.44 ± 0.01	0.52 ± 0.06
cis‐Caryophyllene	1573	n.d.	n.d.	1.70 ± 0.30	0.77 ± 0.00	0.75 ± 0.02
Caryophyllene	1604	4.01 ± 0.22	3.99 ± 0.00	1.09 ± 0.00	1.29 ± 0.02	1.74 ± 0.07
Humulene	1681	n.d.	n.d.	0.13 ± 0.00	n.d.	n.d.
γ‐Bisabolene	1762	0.86 ± 0.05	1.20 ± 0.02	1.80 ± 0.31	n.d.	0.27 ± 0.00
Tot %		7.67	6.43	6.03	2.50	3.28
*Esters*						
Nitric acid, pentyl ester	1220	n.d.	n.d.	0.35 ± 0.07	0.44 ± 0.00	0.34 ± 0.07
Octanoic acid, methyl ester	1387	n.d.	3.70 ± 0.18	0.89 ± 0.00	0.58 ± 0.06	0.51 ± 0.02
Octanoic acid, ethyl ester	1435	10.31 ± 0.03	8.52 ± 0.15	5.20 ± 1.00	1.88 ± 0.21	1.86 ± 0.07
Isobornyl acetate	1582	n.d.	n.d.	0.36 ± 0.07	n.d.	n.d.
Decanoic acid, methyl ester	1600	n.d.	0.51 ± 0.09	n.d.	n.d.	n.d.
Decanoic acid, ethyl ester	1643	n.d.	1.06 ± 0.05	0.51 ± 0.06	n.d.	n.d.
Butanoic acid, 2‐methyl‐	1662	n.d.	n.d.	2.04 ± 0.40	1.57 ± 0.30	n.d.
Tot %		10.31	13.79	9.35	4.47	2.71
*Furans*						
Furan,2‐pentyl‐	1231	1.09 ± 0.04	0.98 ± 0.04	0.71 ± 0.14	0.61 ± 0.00	0.61 ± 0.06
*Ketones*						
Acetoin	1287	n.d.	n.d.	3.63 ± 0.08	0.75 ± 0.00	1.53 ± 0.05
6‐Methyl‐5‐hepten‐2‐one	1339	2.66 ± 0.02	2.41 ± 0.06	4.00 ± 0.70	3.48 ± 0.09	2.93 ± 0.26
2‐Nonanone	1398	n.d.	1.03 ± 0.00	n.d.	n.d.	n.d.
3‐Octen‐2‐one	1414	3.45 ± 0.04	n.d.	n.d.	5.35 ± 0.01	4.66 ± 0.32
3,5‐Octadien‐2‐one	1516	0.55 ± 0.01	0.63 ± 0.05	1.90 ± 0.20	1.30 ± 0.14	1.04 ± 0.01
Geranylacetone	1852	2.57 ± 0.21	2.54 ± 0.08	3.51 ± 0.70	2.34 ± 0.06	2.68 ± 0.17
Tot %		9.23	6.61	13.04	13.22	12.84
*Hydrocarbons*						
Tridecane,3‐methyl‐	1365	n.d.	n.d.	0.52 ± 0.03	n.d.	n.d.
*Organic Acids*						
Acetic acid	1441	5.92 ± 0.21	6.94 ± 0.03	10.84 ± 2.00	10.10 ± 0.52	12.58 ± 0.55
Butanoic acid	1620	n.d.	n.d.	n.d.	n.d.	n.d.
Pentanoic acid	1729	1.18 ± 0.00	1.05 ± 0.02	1.08 ± 0.20	1.21 ± 0.02	1.27 ± 0.16
Hexanoic acid	1834	17.27 ± 0.43	16.62 ± 0.08	18.50 ± 3.50	16.79 ± 0.40	17.90 ± 0.09
Nonanoic acid	2162	1.28 ± 0.51	1.37 ± 0.10	2.40 ± 0.45	1.82 ± 0.35	1.64 ± 0.23
Tot %		25.65	25.98	32.82	29.92	33.39
*Nitriles*						
Benzonitrile	1614	n.d.	n.d.	n.d.	0.20 ± 0.02	0.10 ± 0.00
*Carotenoids*						
β‐Cyclocitral	1622	5.29 ± 0.04	5.21 ± 0.06	5.41 ± 0.56	5.11 ± 0.02	4.32 ± 0.12
α‐lonone	1849	7.55 ± 0.02	7.67 ± 0.45	14.43 ± 2.00	13.44 ± 0.49	13.65 ± 0.23
Tot %		12.84	12.88	19.84	18.55	17.97

**TABLE 7:** Volatile composition (% area) of carrots treated with plasma activated water (PAW).

	R.I.	Control	5 min	10 min	20 min	30 min
*Aldehydes*						
Heptanal	1194	1.28 ± 0.23	2.15 ± 0.02	2.37 ± 0.07	1.83 ± 0.04	1.70 ± 0.14
Octanal	1291	n.d.	4.92 ± 0.00	5.25 ± 0.14	4.56 ± 0.15	4.67 ± 0.45
2‐Heptenal	1332	n.d.	3.00 ± 1.11	3.31 ± 0.01	n.d.	n.d.
Nonanal	1396	5.12 ± 0.00	n.d.	4.28 ± 0.08	n.d.	n.d.
2‐Octenal	1434	n.d.	5.08 ± 0.02	4.70 ± 0.48	4.75 ± 0.41	4.74 ± 0.19
Decanal	1502	n.d.	0.95 ± 0.00	1.01 ± 0.06	0.89 ± 0.06	n.d.
Benzaldehyde	1528	0.89 ± 0.16	0.69 ± 0.04	0.80 ± 0.05	0.79 ± 0.05	3.82 ± 0.00
2‐Nonenal	1537	3.04 ± 0.62	4.05 ± 0.01	4.18 ± 0.04	3.45 ± 0.18	4.36 ± 0.08
Benzeneacetaldehyde	1638	0.87 ± 0.00	0.66 ± 0.00	0.78 ± 0.02	0.98 ± 0.06	0.36 ± 0.00
2,4‐Nonadienal	1705	n.d.	0.80 ± 0.02	0.35 ± 0.00	0.77 ± 0.09	n.d.
Tot %		11.20	22.30	27.03	18.02	19.65
*Alcohols*						
1‐Pentanol	1250	n.d.	n.d.	0.61 ± 0.05	0.80 ± 0.14	0.38 ± 0.00
1‐Octen‐3‐ol	1445	1.54 ± 0.00	2.32 ± 0.05	1.44 ± 0.16	2.04 ± 0.10	n.d.
2,3‐Butanediol	1576	2.05 ± 0.23	1.01 ± 0.00	0.67 ± 0.11	0.58 ± 0.06	0.24 ± 0.00
2‐Octen‐1‐ol	1612	n.d.	n.d.	n.d.	0.20 ± 0.00	n.d.
*p*‐Cymen‐8‐ol	1846	0.37 ± 0.01	0.22 ± 0.51	0.28 ± 0.02	0.38 ± 0.04	n.d.
Tot %		3.96	3.55	3.00	4.00	0.62
*Terpenes*						
Caryophyllene	1604	7.64 ± 0.09	4.49 ± 1.10	3.63 ± 0.24	2.88 ± 0.03	4.18 ± 0.15
γ‐Bisabolene	1762	4.57 ± 0.40	5.00 ± 0.11	6.81 ± 0.45	3.79 ± 0.23	5.58 ± 0.25
Tot %		12.21	9.49	10.44	6.67	9.76
*Esters*						
Octanoic acid, ethyl ester	1435	n.d.	n.d.	n.d.	n.d.	21.83 ± 1.01
Tot %		0.00	0.00	0.00	0.00	21.83
*Furans*						
Furan,2‐pentyl‐	1231	n.d.	n.d.	0.73 ± 0.02	0.62 ± 0.03	0.37 ± 0.00
*Ketones*						
6‐Methyl‐5‐hepten‐2‐one	1339	5.76 ± 0.21	3.71 ± 0.06	3.80 ± 0.13	3.51 ± 0.33	6.22 ± 0.85
3‐Nonanone	1357	2.17 ± 0.42	1.67 ± 0.00	2.14 ± 0.16	1.37 ± 0.02	1.85 ± 0.19
2‐Nonanone	1398	n.d.	n.d.	1.21 ± 0.20	0.45 ± 0.00	0.62 ± 0.00
3,5‐Octadien‐2‐one	1516	n.d.	1.77 ± 0.06	1.69 ± 0.05	1.60 ± 0.01	1.59 ± 0.15
Geranylacetone	1852	6.43 ± 0.00	2.36 ± 0.01	2.69 ± 0.11	3.92 ± 0.38	4.20 ± 0.71
Tot %		14.36	9.51	11.53	10.85	14.48
*Organic Acids*						
Acetic acid	1441	34.06 ± 0.62	22.86 ± 1.02	21.86 ± 0.98	27.75 ± 3.05	1.07 ± 0.00
Butanoic acid	1620	n.d.	0.08 ± 0.51	0.31 ± 0.05	0.24 ± 0.00	n.d.
Pentanoic acid	1729	n.d.	0.62 ± 0.00	0.30 ± 0.00	0.38 ± 0.00	n.d.
Hexanoic acid	1834	1.55 ± 0.05	7.46 ± 0.35	6.35 ± 0.27	3.47 ± 0.00	5.61 ± 0.04
Octanoic acid	2070	n.d.	1.19 ± 0.24	1.57 ± 0.31	1.45 ± 0.14	0.50 ± 0.70
Nonanoic acid	2162	6.71 ± 1.24	3.12 ± 1.32	2.17 ± 0.20	3.67 ± 0.61	3.25 ± 0.64
Tot %		42.32	35.33	32.56	36.96	10.43
*Carotenoids*						
β‐Cyclocitral	1622	4.43 ± 0.10	4.73 ± 1.11	4.54 ± 0.38	4.39 ± 0.56	4.95 ± 0.10
α‐lonone	1849	9.07 ± 0.08	7.66 ± 0.00	8.01 ± 0.04	9.91 ± 0.86	9.56 ± 0.26
Tot %		13.50	12.39	12.55	14.30	14.51

The aldehydes in CP treated carrots had an oscillatory trend: in fact, the total aldehydes content (%) increased after 5 min (7%), then decreased after 10–20 min (9%–4%, respectively), and finally increased after 30 min (8%). The volatiles mainly involved in these changes were: heptanal, 2‐hexenal, octanal, 2‐heptenal and 2‐octenal; while the only ones that showed an increase were nonanal and 2,4‐nonadienal. The same class showed an opposite behavior in PAW‐treated samples: in fact, the highest increment was found in carrots treated for 10 min (60%), while the lowest one in those treated for 30 min (44%). This result was reported in the literature, in fact an increase in total aldehydes was observed after 5 min of PAW treated rocket samples (Abouelenein and Vittori 2021). The increase in aldehydes after PAW treatment is likely to be due to the degradation of unsaturated fatty acids, triggered by the reactive species of the plasma, which lead to the formation of secondary oxidation products, such as aldehydes (Benedetti and Esterbauer 1984; Mead 1976).

The volatiles mainly involved in these changes were: heptanal, benzaldehyde and 2‐nonenal, while benzene acetaldehyde showed an opposite behavior, as it decreased from 0.87% to 0.36% and showed an increase only in the sample treated for 20 min. Aldehydes in carrots and vegetables are responsible for green, leafy, fatty, and sweet undertones (Tian and Wang 2024).

The abundance of alcohols, known for their earthy, fresh aroma profile, slightly increased after 5 min of CP treatment (10%) and then decreased with increasing treatment time. Alcohols in the samples treated with PAW decreased, especially after 30 min of treatment (85%).

Terpenes in the CP‐treated samples decreased as the treatment time increased, especially after 20 min of treatment (68%). In contrast, for PAW‐treated samples, an oscillatory trend in their abundance was observed, with an overall decrease along with the increment of treatment time, with the lowest value being observed in the carrots treated for 20 min (46%). The most abundant terpenes in CP and PAW‐treated samples were caryophyllene and γ‐bisabolene, known for their terpene‐like, spicy, woody and balsamic citrus myrrh tones, respectively.

The abundance of esters in CP‐treated samples increased after 5 min of treatment (26%), then decreased proportionally with increasing treatment duration. In contrast, octanoic acid, ethyl ester was present only in the sample treated with PAW for 30 min.

Among the furans, only furan, 2‐pentyl was identified, which decreased in the CP treated carrots, while in PAW‐treated carrots it was present only after 10 min of treatment and then decreased after 20 and 30 min of treatment.

Ketones in the CP‐treated samples decreased after 5 min of treatment (29%), then increased with treatment time, especially in carrots treated for 10 min (30%). The abundance of ketones decreased with treatment time by 34‐20‐25%, while it increased by 1% after 30 min of treatment.

Organic acids had an overall increase in the CP‐treated samples, especially in the carrots treated for 30 min (24%), while their abundance decreased in the PAW‐treated samples as time treatment increased, especially after 30 min of treatment (76%).

Carotenoids increased in the CP‐treated samples, especially in the carrots treated for 10 min (36%), while their abundance increased in the PAW‐treated samples after 20–30 min (6%–7%). The only two carotenoids present in both CP and PAW‐treated samples were β‐cyclocitral and α‐ionone, which have mint and wood/violet odors, respectively.

In general, the abundance of volatiles was slightly increased with the application of CP treatment to carrots, with the highest increase observed in the carrots treated for 10 min (10%). On the contrary, the abundance of volatiles was slightly increased (1%) in carrots treated with PAW for 10 min, while in carrots treated for 5‐20‐30 min it decreased by 6‐7‐7%, respectively.

In conclusion, the application of both CP and PAW for 10 min slightly increased the amount of volatile compounds in fresh‐cut carrots.

### 3.5 Spectrophotometrical Analysis

The results of TPC, TFC and DPPH are shown in Tables 8 and 9. TPC showed an increase in the total content of polyphenols up to 20 min of treatment, while it decreased after 30 min. In the PAW‐treated carrots, the TPC value showed an oscillatory trend, with the highest value found in carrots treated for 5 min and the lowest one after 10 min.

**TABLE 8:** Spectrophotometric analyses (TPC, TFC, and DPPH) in CP treated carrots.

CP	Control	5 min	10 min	20 min	30 min
TPC (mg GAE/Kg DE)	26.47 ± 0.9^a^	87.56 ± 1.4^b^	297.26 ± 0.3^c^	314.82 ± 0.5^d^	65.11 ± 3.8^e^
TFC (mg RE/Kg DE)	2248.48 ± 11.9^a^	2327.92 ± 3.5^b^	2098.24 ± 10.9^c^	1701.33 ± 6.5^d^	1949.31 ± 1.8^e^
DPPH (mgTE/Kg DE)	155.49 ± 0.2^a^	208.15 ± 1.3^b^	180.00 ± 0.5^c^	134.60 ± 1.3^d^	159.47 ± 2.7^e^

*Note:* Different letters indicate statistically significant differences (*p* < 0.05) among samples with the same treatment (*n* = 3).

**TABLE 9:** Spectrophotometric analyses (TPC, TFC, and DPPH) in PAW treated carrots.

PAW	Control	5 min	10 min	20 min	30 min
TPC (mg GAE/Kg DE)	161.68 ± 0.6^a^	238.14 ± 0.9^b^	106.03 ± 0.8^c^	201.68 ± 0.6^d^	176.51 ± 9.6^e^
TFC (mg RE/Kg DE)	576.43 ± 8.0^a^	463.91 ± 3.6^b^	640.08 ± 2.7^c^	690.25 ± 1.7^d^	686.98 ± 8.7^d^
DPPH (mgTE/Kg DE)	138.96 ± 1.9^a^	77.39 ± 6.7^b^	27.22 ± 2.7^c^	152.94 ± 1.4^a^	35.16 ± 2.2^c^

*Note:* Different letters indicate statistically significant differences (*p* < 0.05) among samples with the same treatment (*n* = 3).

The highest TFC value was found in carrots treated with CP for 5 min and with PAW for 20 min. Similarly, antioxidant activity (DPPH) of carrots increased after 5 min of CP and after 20 min of PAW.

These results were not consistent with those obtained in the quantitative analysis of polyphenols by HPLC‐MS/MS. This can be explained by the fact that spectrophotometric analyses are not as specific as a quantitative analysis because other molecules (such as other reducing compounds) can interact with the reagents used and alter results (Huang et al. 2005; Everette and Walker 2010), as well as interferences from oxidation products.

However, quantitative analysis by HPLC‐MS/MS provides clearer data and is difficult to misunderstand, as it uses methods that are validated with analytical standards and allow to quantify the molecules of interest.

### 3.6 Principal Component Analysis of Volatile Profiles

Principal Component Analysis (PCA) was applied to study the possible correlation between the volatile profile and the treatment time of the analyzed samples.

Regarding carrots treated with CP (Figure 3), the results highlighted that the samples treated for 10 min differ the most from the control. In the same way, carrots treated with CP for 5 min resulted to have an opposite volatile profile with respect to those treated with CP for 20 and 30 min.

**FIGURE 3:**
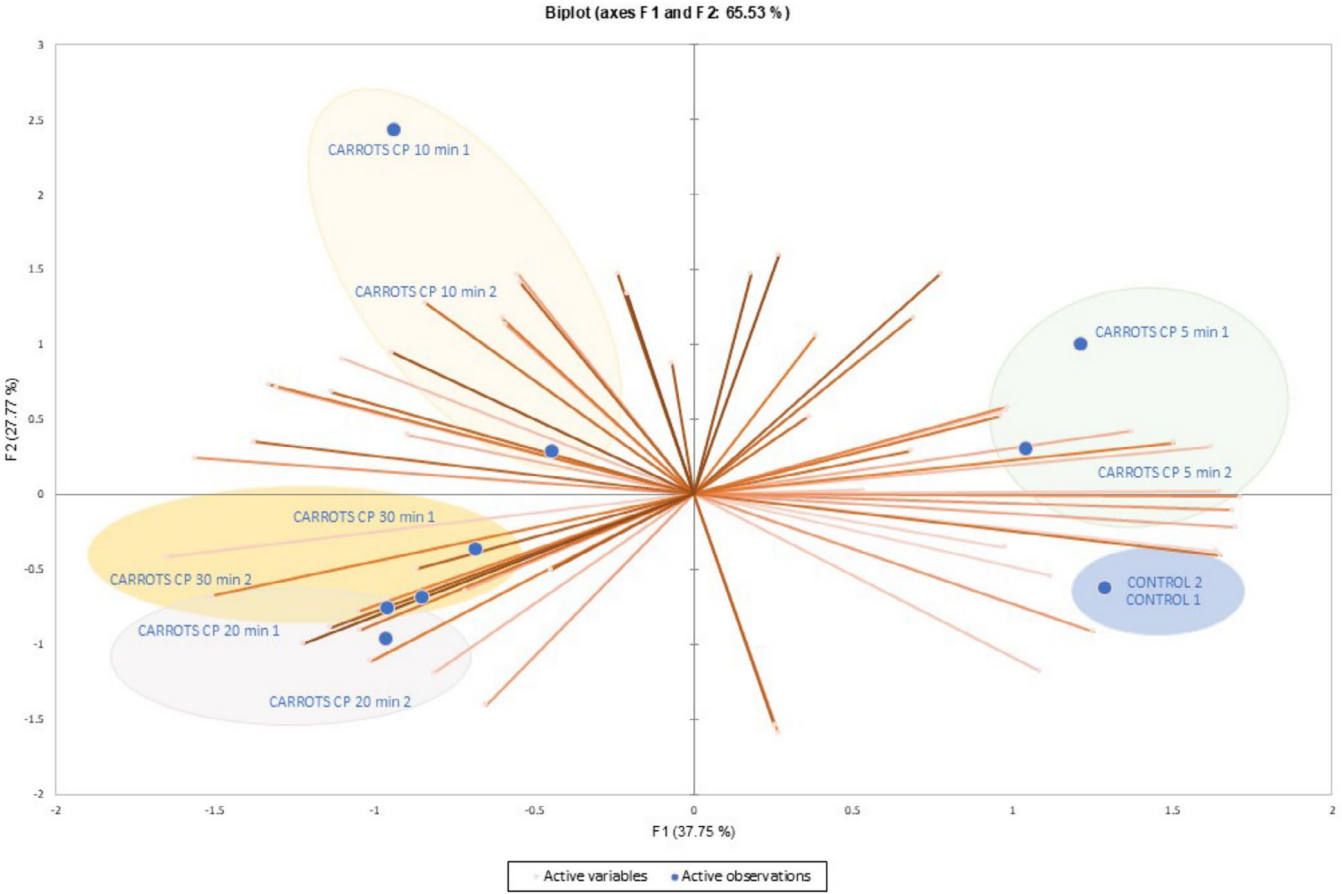
PCA of volatile compounds in CP‐treated carrots.

By integrating these results with those obtained by HS‐SPME‐GC–MS analysis, it appears that the 10 min CP treatment completely changed the volatile profile, even when the abundance of volatile compounds was increased. The 5 min CP treatment resulted in a slight increase in the abundance of volatile compounds and it better preserved the volatile composition compared to the control.

The PCA revealed that the PAW treatment of the carrots completely influenced the volatile profile (Figure 4). Treatment duration, instead, had no effect on the volatile composition, especially for samples treated for 5‐10‐20 min. In general, the results of HS‐SPME‐GC–MS analysis revealed that the abundance of volatiles increased slightly after 10 min of treatment, but the composition of volatiles changed completely during PAW treatment.

**FIGURE 4:**
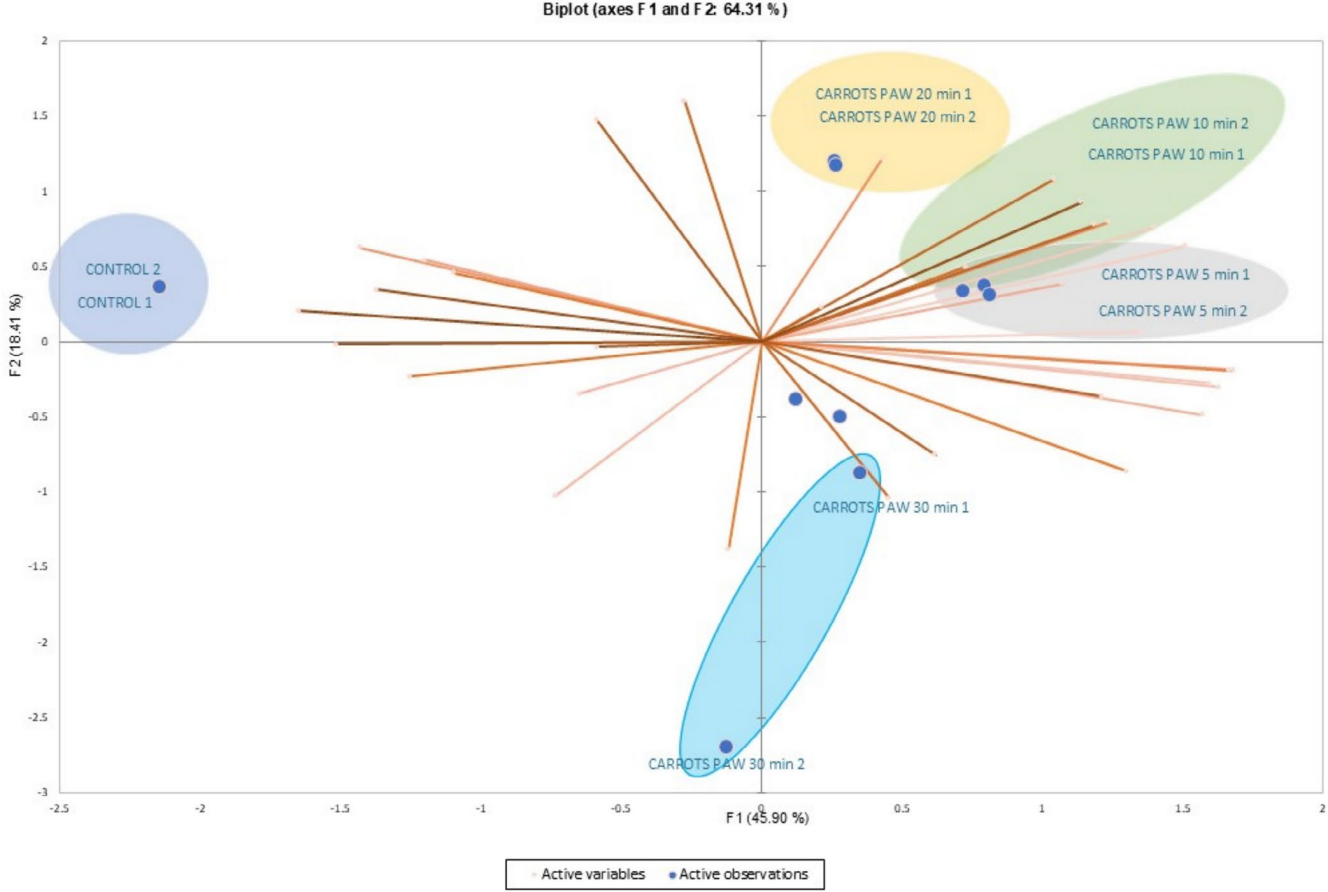
PCA of volatile compounds in PAW‐treated carrots.

## 4 Conclusions

This study showed that CP and PAW treatments affect the chemical composition of fresh‐cut carrots. The quantitative analysis of polyphenols by HPLC‐MS/MS showed a significant decrease in the total content of polyphenols in the carrots treated with PAW, while in carrots treated with CP the total content of polyphenols remained almost unchanged, with no statistically significant changes.

Ascorbic acid in carrots was affected by CP and PAW treatments: in CP‐treated carrots, it decreased with increasing treatment time, with statistically significant differences up to 10 min of treatment. In contrast, in PAW‐treated carrots, the concentration of ascorbic acid increased with 5–10 min of treatment and then dropped slightly after 20 min and rose again after 30 min, but without statistically significant differences.

Regarding vitamins B2 and B3, riboflavin was completely absent in all samples, while nicotinamide decreased in both PAW‐treated and CP‐treated samples. On the contrary, nicotinic acid showed a significant increase in CP treated samples compared to the PAW treated ones, indicating a possible conversion of nicotinamide into nicotinic acid.

The analysis of volatiles showed that both treatments resulted in changes in the abundance of aldehydes, alcohols, terpenes, esters, furans, ketones, hydrocarbons and organic acids. In general, as exposure time increased, a reduction of some compounds and an increase in others was observed, suggesting that the treatment affects the chemical reactions underlying the production of these substances. PCA revealed that a 5 min CP treatment slightly increased the abundance of volatile compounds preserving the volatile composition compared to the control, while the PAW treatment completely changed it.

The spectrophotometric analyses of the CP and PAW cores differed from the quantitative analysis of the polyphenols. In fact, the highest TPC value is found with carrots treated with CP for 20 min and with PAW for 5 min; on the other hand, ì the TFC shows the highest values in carrots treated with CP for 5 min and with PAW for 20 min; finally, the antioxidant activity, measured by the DPPH test, was higher in carrots treated with CP for 5 min and with PAW for 20 min.

In conclusion, this study shows that both CP and PAW treatments contribute to altering the chemical composition of fresh‐cut carrots, with PAW treatment having a greater impact on the content of bioactive compounds. In particular, PAW treatment negatively affect the chemical composition, especially polyphenols concentration; while ascorbic acid, nicotinic acid and nicotinamide remained invariable. CP treatment seemed to better maintain the chemical composition of carrots, in terms of both bioactive molecules and volatile compounds.

This study has thus shown that not only the treatment, but also the matrix is of fundamental importance; in fact, the matrix, interacting with the different species present in the CP and the PAW, also influences the content of various bioactive compounds.

New studies are needed to better understand the interactions between the plasma reactive species and the food matrices subjected to these treatments, in order to exploit the potential of these non‐thermal technologies without altering the nutritional properties of the food.

## Author Contributions

**L. Acquaticci:** conceptualization, writing – original draft. **E. Torregiani:** methodology, software, data curation. **J. B. Molina Hernandez:** investigation, validation, formal analysis. **G. G. Gebremical:** visualization, formal analysis, supervision. **S. Tappi:** writing – review and editing, project administration. **G. Caprioli:** writing – review and editing, project administration, resources.

## Funding

The project “TECH4PATH—Non‐thermal TECHnologies FOR the inactivation of emerging viral, bacterial and protozoan PATHogens on fruit and vegetable products” is founded by MUR—Ministero dell'Università e della Ricerca—PRIN: Progetti di Ricerca di Rilevante Interesse Nazionale, Bando 2022.

## Conflicts of Interest

The authors declare no conflicts of interest.

## Supporting information


**Table S1:** HPLC–MS/MS acquisition parameters (dynamic‐MRM mode) used for the analysis of the 36 polyphenols.
**Table S2:** High performance liquid chromatography/tandem mass spectrometry (HPLC–MS/MS) acquisition parameters (multiple reaction monitoring mode, MRM) used for the analysis of the target analytes.

## Data Availability

The data that support the findings of this study are available from the corresponding author upon reasonable request.
