# Supplementary material for: Effect of Cold Plasma and Plasma Activated Water on Polyphenols, Vitamins, and Volatile Compounds in Fresh‐Cut Carrots
Source: Food Sci Nutr. 2026 Oct 29;14(11):e72526. doi: 10.1002/fsn3.72526 (PMC13719803; doi:10.1002/fsn3.72526)
Supplement: Supplementary file 1 [file FSN3-14-e72526-s001.docx]

**Table S1.** HPLC–MS/MS acquisition parameters (dynamic-MRM mode) used for the analysis of the 36 polyphenols.

| **No.** | **Compounds** | **Precursor ion, *m*/*z*** | **Product ion, *m*/*z*** | **Fragm-entor, V** | **Collision energy, V** | **Polarity** | **Retention time (Rt, min)** |
| --- | --- | --- | --- | --- | --- | --- | --- |
| 1 | Gallic acid | 169 | 125.2[^*^](https://www.sciencedirect.com/science/article/pii/S0308814621017490#tblfn1) | 97 | 12 | Negative | 6.96 |
| 2 | Neochlorogenic acid | 353 | 191.2[^*^](https://www.sciencedirect.com/science/article/pii/S0308814621017490#tblfn1), 179 | 82 | 12, 12 | Negative | 9.52 |
| 3 | Delphinidin-3-galactoside | 465.01 | 303[^*^](https://www.sciencedirect.com/science/article/pii/S0308814621017490#tblfn1) | 121 | 20 | Positive | 11.36 |
| 4 | (+)-Catechin | 289 | 245.2[^*^](https://www.sciencedirect.com/science/article/pii/S0308814621017490#tblfn1),109.2 | 131 | 8, 20 | Negative | 11.44 |
| 5 | Procyanidin B2 | 576.99 | 576.99[^*^](https://www.sciencedirect.com/science/article/pii/S0308814621017490#tblfn1), 321.2 | 160 | 0, 32 | Negative | 12.41 |
| 6 | Chlorogenic acid | 353 | 191.2[^*,^](https://www.sciencedirect.com/science/article/pii/S0308814621017490#tblfn1) 127.5 | 82 | 12, 20 | Negative | 12.42 |
| 7 | *p*-Hydroxybenzoic acid | 137 | 93.2[^*^](https://www.sciencedirect.com/science/article/pii/S0308814621017490#tblfn1) | 92 | 16 | Negative | 12.86 |
| 8 | (−)-Epicatechin | 289 | 245.1[^*^](https://www.sciencedirect.com/science/article/pii/S0308814621017490#tblfn1), 109.1 | 126 | 8, 20 | Negative | 13.03 |
| 9 | Cyanidin-3-glucoside | 449 | 287.3[^*^](https://www.sciencedirect.com/science/article/pii/S0308814621017490#tblfn1), 255.6 | 121 | 20, 20 | Positive | 13.14 |
| 10 | Petunidin-3-glucoside | 479.01 | 317[^*^](https://www.sciencedirect.com/science/article/pii/S0308814621017490#tblfn1), 302 | 121 | 20, 44 | Positive | 13.26 |
| 11 | 3-Hydroxybenzoic acid | 137 | 93.2[^*^](https://www.sciencedirect.com/science/article/pii/S0308814621017490#tblfn1) | 88 | 8 | Negative | 13.59 |
| 12 | Caffeic acid | 179 | 135.2[^*^](https://www.sciencedirect.com/science/article/pii/S0308814621017490#tblfn1), 134.1 | 92 | 12, 24 | Negative | 13.65 |
| 13 | Vanillic acid | 167 | 152.4[^*^](https://www.sciencedirect.com/science/article/pii/S0308814621017490#tblfn1), 108.1 | 88 | 12, 20 | Negative | 14.32 |
| 14 | Pelargonidin-3-glucoside | 433.01 | 271[^*^](https://www.sciencedirect.com/science/article/pii/S0308814621017490#tblfn1), 121 | 116 | 24, 50 | Positive | 14.52 |
| 15 | Pelagonidin-3-rutinoside | 579.01 | 271[^*^](https://www.sciencedirect.com/science/article/pii/S0308814621017490#tblfn1) | 145 | 32 | Positive | 14.56 |
| 16 | Malvidin-3-galactoside | 493.01 | 331[^*^](https://www.sciencedirect.com/science/article/pii/S0308814621017490#tblfn1), 315.1 | 121 | 20, 50 | Positive | 14.64 |
| 17 | Syringic acid | 196.9 | 182.2[^*^](https://www.sciencedirect.com/science/article/pii/S0308814621017490#tblfn1), 121.2 | 93 | 8, 12 | Negative | 15.28 |
| 18 | Procyanidin A2 | 575 | 575[^*^](https://www.sciencedirect.com/science/article/pii/S0308814621017490#tblfn1), 285 | 170 | 0, 20 | Negative | 16.18 |
| 19 | *p*-Coumaric acid | 163 | 119.2[^*^](https://www.sciencedirect.com/science/article/pii/S0308814621017490#tblfn1), 93.2 | 83 | 12, 36 | Negative | 16.70 |
| 20 | Ferulic acid | 193 | 134.2[^*^](https://www.sciencedirect.com/science/article/pii/S0308814621017490#tblfn1), 131.6 | 83 | 12, 8 | Negative | 17.10 |
| 21 | 3,5-Dicaffeoylquinic acid | 514.9 | 353.1[^*^](https://www.sciencedirect.com/science/article/pii/S0308814621017490#tblfn1), 191 | 117 | 8, 28 | Negative | 17.61 |
| 22 | Rutin | 609 | 300.2[^*^](https://www.sciencedirect.com/science/article/pii/S0308814621017490#tblfn1), 271.2 | 170 | 32, 50 | Negative | 17.73 |
| 23 | Hyperoside | 465.01 | 303[^*^](https://www.sciencedirect.com/science/article/pii/S0308814621017490#tblfn1), 61.1 | 97 | 8, 50 | Positive | 18.33 |
| 24 | Isoquercitrin | 463 | 271.2[^*^,](https://www.sciencedirect.com/science/article/pii/S0308814621017490#tblfn1) 300.2 | 155 | 44, 24 | Negative | 18.36 |
| 25 | Delphinidin-3,5-diglucoside | 462.9 | 300.1[^*^](https://www.sciencedirect.com/science/article/pii/S0308814621017490#tblfn1) | 165 | 24 | Negative | 18.38 |
| 26 | Phloridzin | 435.39 | 273[^*^](https://www.sciencedirect.com/science/article/pii/S0308814621017490#tblfn1), 167 | 155 | 8, 28 | Negative | 18.83 |
| 27 | Quercitrin | 446.99 | 300.2[^*^,](https://www.sciencedirect.com/science/article/pii/S0308814621017490#tblfn1) 301.2 | 160 | 24, 16 | Negative | 19.61 |
| 28 | Myricetin | 316.99 | 179.1[^*^,](https://www.sciencedirect.com/science/article/pii/S0308814621017490#tblfn1) 182 | 150 | 16, 24 | Negative | 19.61 |
| 29 | Naringin | 578.99 | 271.3[^*^,](https://www.sciencedirect.com/science/article/pii/S0308814621017490#tblfn1) 151.3 | 170 | 32, 44 | Negative | 19.62 |
| 30 | Kaempferol-3-glucoside | 447 | 284.2[^*^,](https://www.sciencedirect.com/science/article/pii/S0308814621017490#tblfn1) 255.2 | 170 | 24, 40 | Negative | 19.77 |
| 31 | Hesperidin | 611.01 | 303[^*^,](https://www.sciencedirect.com/science/article/pii/S0308814621017490#tblfn1) 334.8 | 112 | 20, 12 | Positive | 20.19 |
| 32 | Ellagic acid | 301 | 301[^*^](https://www.sciencedirect.com/science/article/pii/S0308814621017490#tblfn1), 229 | 170 | 0, 24 | Negative | 21.41 |
| 33 | Quercetin | 300.99 | 151.2[^*^,](https://www.sciencedirect.com/science/article/pii/S0308814621017490#tblfn1) 179.2 | 145 | 16, 12 | Negative | 21.87 |
| 34 | Phloretin | 272.99 | 167[^*^,](https://www.sciencedirect.com/science/article/pii/S0308814621017490#tblfn1) 123 | 116 | 8, 20 | Negative | 22.30 |
| 35 | Kaempferol | 287.01 | 153[^*^](https://www.sciencedirect.com/science/article/pii/S0308814621017490#tblfn1), 69.1 | 60 | 36, 50 | Positive | 23.84 |
| 36 | Isorhamnetin | 314.99 | 300.2[^*^,](https://www.sciencedirect.com/science/article/pii/S0308814621017490#tblfn1) 196.1 | 145 | 16, 4 | Negative | 24.57 |

*These product ions were used for quantification; the rest of the product ions were used for identification.

**Table S2.** High performance liquid chromatography/tandem mass spectrometry (HPLC–MS/MS) acquisition parameters (multiple reaction monitoring mode, MRM) used for the analysis of the target analytes.

| **Compounds** | **Retention Time (min)** | **Precursor ion (*m*/*z*)** | **Product ion (*m*/*z*)** | **Fragmentor (V)** | **Collision energy** | **Polarity** |
| --- | --- | --- | --- | --- | --- | --- |
| Nicotinic acid | 1.59 | 124 | 80.1^a^ | 92 | 21 | Positive |
|  |  |  | 53.2 |  | 33 |  |
| Nicotinamide | 1.72 | 123 | 80.1^b^ | 92 | 21 | Positive |
|  |  |  | 53.2 |  | 33 |  |
| Riboflavin | 2.12 | 377 | 243.1^c^ | 131 | 21 | Positive |
|  |  |  | 172.1 |  | 41 |  |

^a,b,c^ These product ions were used for the quantification; the rest of the product ions were used for identification.
